# A Recombinant RBD-Based Phage Vaccine Report: A Solution to the Prevention of New Diseases?

**DOI:** 10.3390/vaccines11040833

**Published:** 2023-04-13

**Authors:** Zahra Salehi, Mohammad Javad Rasaee

**Affiliations:** Department of Medical Biotechnology, Tarbiat Modares University, Tehran 1411713116, Iran

**Keywords:** phage vaccine, COVID-19, SARS-CoV-2, spike protein

## Abstract

The safety, inherent immunogenicity, stability, and low-cost production of bacteriophages make them an ideal platform for vaccine development. Most vaccination strategies against COVID-19 have targeted the spike protein of SARS-CoV-2 to generate neutralizing antibodies. P1, a truncated RBD-derived spike protein, has been shown to induce virus-neutralizing antibodies in preclinical studies. In this study, we first investigated whether recombinant phages displaying P1 on the M13 major protein could immunize mice against COVID-19, and second, whether inoculation with 50 µg of purified P1 in addition to the recombinant phages would stimulate the immune systems of the animals. The results showed that the mice that received recombinant phages were immunized against the phage particles, but did not have anti-P1 IgG. In contrast, compared with the negative control, the group that received a combination of P1 protein and recombinant phage was immunized against the P1 protein. In both groups, CD4^+^ and CD8^+^ T cells appeared in the lung tissue. These results suggest that the number of antigens on the phage body plays a crucial role in stimulating the immune system against the bacteriophage, although it is immunogenic enough to function as a phage vaccine.

## 1. Introduction

After the first report of mysterious pneumonia in Wuhan in December 2019, which subsequently spread worldwide [1], there was an urgent need to control pathogen transmission and mortality. This led to the development of 172 and 199 vaccine candidates in the clinical and preclinical phases, respectively, based on the WHO (World Health Organization) vaccine tracker [2]. Of these, nine candidates received emergency use authorization (EUA) and were evaluated by the Food and Drug Administration (FDA) as safe and effective for human administration [3]. Vaccine platforms for COVID-19 (Coronavirus Disease 2019), caused by SARS-CoV-2 (Severe Acute Respiratory Syndrome Coronavirus 2) [4], ranged from inactivated virus, protein subunit, and replicative and non-replicative viral vectors to DNA, mRNA, and virus-like particles (VLPs) [2,5]. Nevertheless, COVID-19 vaccination could reduce the rate of disease symptoms, hospitalizations, and deaths [6,7]. Research into alternative vaccination strategies to combat emerging outbreaks is ongoing because the need for a thermostable vaccine with long-lasting protection and the potential for large-scale production has not yet been met [8].

The era of bacteriophage-based vaccines dates back to 1988, when de la Cruz’s research team produced a recombinant f1 filamentous phage containing the circumsporozoite protein of Plasmodium falciparum (the causative agent of malaria), which was shown to be antigenic and immunogenic in rabbits [9]. Bacteriophages would be an ideal vaccine platform because their administration to humans is safe, their production process is cost-effective and easy to perform on a large scale, they have intrinsic adjuvant properties and immunogenicity, and they can withstand harsh thermal and pH conditions [9,10]. Phages are widely used in phage therapy, epitope mapping antibody recognition, drug and gene discovery, tissue engineering, and biosensor development. Phage-based vaccines have been used to treat cancer as well as viral, bacterial, fungal, and parasitic infectious diseases [11,12,13]. It is worth mentioning that in the case of the COVID-19 pandemic, researchers have not overlooked the phage-based platform, and have demonstrated its efficacy and usefulness as a vaccine candidate in preclinical studies [9,14,15,16,17,18].

The spike (S) glycoprotein of SARS-CoV-2 is the main target of vaccines because it plays a key role in the entry of the virus into host cells. It consists of subunits S1 and S2, with S1 involved in host receptor ACE2 (angiotensin-converting enzyme 2) recognition and binding through its RBD (receptor-binding domain), whereas S2 is responsible for viral cell membrane fusion [19,20].

P1, a truncated spike RBD-based 101 amino acid protein, was selected based on in silico analysis tools. It is located in the S1 subunit of the spike protein and contains the entire receptor binding motif (RBM). This protein was found to be expressed in *E. coli* BL21 by Noorabad Ghahroodi F. et al. The optimal concentrations of P1 protein to be injected into mice and rabbits in combination with Freund’s complete adjuvant to generate antibodies were 50 and 350 micrograms, respectively. The results of the ELISA (enzyme-linked immunosorbent assay) tests confirmed the immunogenicity of P1 in both rabbits and mice without lethal consequences. Moreover, this protein interacted with antibodies produced in COVID-19 patients. Antibodies produced by P1 in rabbits were able to successfully neutralize cultured live viruses in the virus neutralization test (VNT), as reported in that study [21].

In this study, we used two strategies to investigate the phage vaccine immunogenicity and adjuvant capacity: 1. We prepared a recombinant M13 phage with P1 on the major phage protein VIII and used it for an immunization study in mice; and 2. We injected 50 micrograms of the purified P1 protein, along with the recombinant and helper phage, into two groups of mice and compared the immunization results.

## 2. Materials and Methods

### 2.1. P1 Expression and Purification

We obtained E. coli BL21 (DE3), transformed with the cloned expression vector PET-28a-P1, from Noorabad Ghahroodi F. et al. [21]. The process of expression, purification, and confirmation of P1 was based on the protocol published by this research group. Briefly, expression of P1 by the transformed BL21 bacteria was achieved by adding 1 mM IPTG (isopropyl β-D-1-thiogalactopyranoside) to the LB culture medium, followed by shaking at 37 °C for 4 h. Then, a Nickle column was used for protein purification, and finally, denaturation and renaturation of P1 were performed by protein dialysis at different urea concentrations and pH values.

Protein size and identity were confirmed by standard 15% *w*/*v* polyacrylamide gel electrophoresis SDS-PAGE (sodium dodecyl-sulfate polyacrylamide gel electrophoresis) and Western blot analysis with anti-His-Tag monoclonal antibodies [21].

### 2.2. Subcloning of the P1 Gene into the pAK8 Phagemid

**Figure 1:**
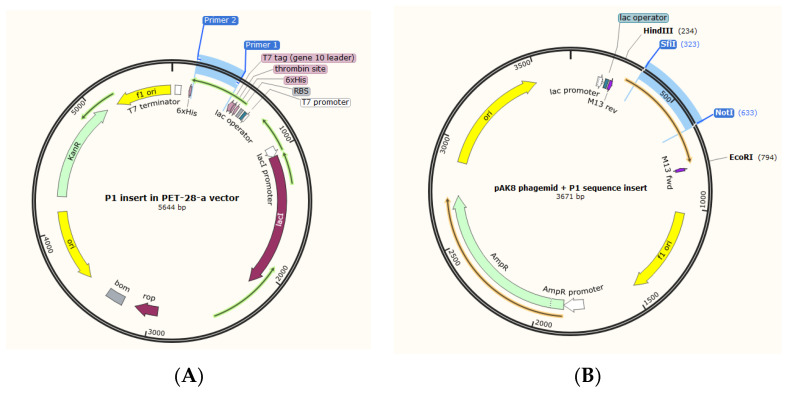
Subcloning of the P1-encoding nucleotide sequence from the PET-28-a to the pAK8 phagemid. (**A**) PET-28-a-P1 vector map. The P1-encoding gene was amplified by PCR using primers with SfiI and NotI restriction enzyme overhangs. (**B**) Map of pAK8-P1 phagemid. Insertion of the amplified gene into the pAK8 phagemid with SfiI and NotI restriction sites. Maps were generated using SnapGene 5.3.1 software.

The P1 gene was amplified from the PET-28a-P1 vector using a primer pair with the SfiI and NotI recognition site overhangs at the 5’ and 3’ ends (Figure 1A). Subsequently, the desired gene was inserted into a pAK8 phagemid (kindly provided by Dr. Alexander Klimka) containing the SfiI and NotI restriction enzyme sites (Figure 1B). The pAK8-P1 construct was then transformed into competent E. coli K12 TG1 cells using the heat-shock protocol, as described elsewhere [22]. Finally, insertion of the P1 gene into the phagemid was confirmed by colony PCR on positive clones with universal M13 primers, followed by restriction mapping and Sanger sequencing of the purified pAK8-P1 construct.

### 2.3. Construction of Recombinant M13 Phages Displaying P1 on pVIII

The production of recombinant phages displaying P1 followed the protocol described by Wu Y. et al. [23]. Briefly, transformed TG1 cells were cultured in 2XYT media until the mid-log phase (OD_600_: 0.5) was reached. Then, these bacteria were infected with helper phages (ratio of bacteria to M13K07 helper phages was 1:20) and grown overnight, with shaking at 25 °C. After that, the bacteria were pelleted by centrifugation, and the recombinant phages were precipitated by adding PEG-NaCl solution (20% polyethylene glycol 6000 with NaCl 2.5 M) to the supernatant. Finally, the pellet was resuspended in PBS buffer and titrated using the serial dilution method.

### 2.4. Detection of P1 on M13 Bacteriophages

Two different ELISA methods were used to confirm the display of P1 as M13 pVIII fusion protein: 1. Sandwich ELISA: Two micrograms of anti-P1 polyclonal antibody, purified by affinity chromatography on a protein A agarose column from immunized rabbit serum (kindly provided by Dr. Faezeh Noorabad Ghahroodi), were coated in each well and then blocked with 5% skimmed milk. Then, 50 µL of 10^10^ pfu/mL recombinant M13 phage and control phage were added to each well in duplicate. M13KO7 helper phage was used as a negative control, as it is a derivative of the M13 bacteriophage and contains all the proteins of the M13 bacteriophage (the recombinant phage) except the P1 protein. After 1 h of incubation, all wells were washed 5 times with PBST and PBS buffers, and 50 µL of anti-M13-HRP (horseradish peroxidase) conjugated monoclonal antibody (1:4000) was added to each well and incubated for 1 h. Plates were then washed, and 50 µL of TMB (3, 3’, 5, 5’-tetramethylbenzidine) was added. The reaction was stopped after 10 min with 50 µL HCl 2N. 2. Indirect ELISA: 10^10^ pfu/mL of recombinant and helper phages were coated in each well. One microgram of purified P1 protein was also coated as a positive control. Then, the purified anti-P1 antibody was added and diluted at a ratio of 1:4000. An anti-rabbit monoclonal antibody (HRP-conjugated) was used at a ratio of 1:30,000. After one hour of incubation, TMB was added and the reaction was stopped using the same protocol. All results were read at a wavelength of 450 nm using a spectrophotometer. The ELISA tests were repeated 3 times.

### 2.5. Analysis of the Interaction of Phage P1 with ACE2

We modeled the 157 amino acid P1-pVIII fusion proteins using in silico modeling servers, and selected the best model based on the QMEAN and Ramachandran plot scores. This model was docked to the ACE2 protein model on the PDB website (PDB ID: 6M0J). On the other hand, a sandwich ELISA was performed experimentally, as described in Section 2.4, to compare the prediction results of the affinity modeling with the actual experimental results. For this analysis, the wells were coated with purified ACE2 protein, which was provided by Pourya Nasirmoghadas. Recombinant P1 phages, control phages, and PBS were then added to each well, the wells were washed, and anti-M13 monoclonal antibodies were added to detect the interacting phages.

### 2.6. Immunization of Animals against Recombinant Phages

Six- to eight-week-old female BALB/c mice (5 mice/per group) intraperitoneally received 200 µL of the following antigens: 10^10^ pfu/mL recombinant phage, 10^10^ pfu/mL helper phage, PBS buffer, 50 µg P1 with 10^10^ pfu/mL recombinant phage, 50 µg P1 with 10^10^ pfu/mL helper phage, and 50 µg P1 with alum as adjuvant. All groups were boosted 2 weeks after the first injection. Blood samples were collected 2 weeks after boosting.

### 2.7. Analysis of Anti-Phage Immunization of Animals

An indirect ELISA was performed to test whether anti-phage antibodies were produced in phage-inoculated groups. We coated 10^10^ pfu/mL of the recombinant phage and helper phage in ELISA 96-well plates. All mice serum samples were diluted to 1:500. An HRP-conjugated mouse monoclonal antibody was used as the secondary antibody at a dilution ratio of 1:15,000.

### 2.8. Analysis of Anti-P1 Immunization of Animals in P1+ Recombinant/Helper Phage Groups

The ELISA procedure was the same as in Section 2.7, but in this experiment, the ELISA wells were coated with 1 µg of P1 protein instead of phage.

Then, in addition to P1, nonspecific proteins P2 and P3 (truncated proteins from other parts of the spike protein) [21], N (SARS-CoV-2 truncated nucleocapsid phosphoprotein) [24], and BSA (bovine serum albumin) were coated in another ELISA assay to test the specificity of the antibodies’ interactions. Pooled serum from each group was used here.

### 2.9. CD4^+^ & CD8^+^ T Cell Analysis by IHC

To assess cell-mediated immunity, lung tissue samples from the mice were examined for the presence of T CD4^+^ and T CD8^+^ by immunohistochemistry (IHC). Animals were sacrificed 1 month after the last injection. Formalin-fixed lung tissue from the mice was embedded in paraffin and then cut into thin 4 µm sections. Next, the primary anti-CD4/CD8 monoclonal antibodies were used. After that, CD4^+^/CD8^+^ T lymphocytes were detected using a Master Polymer Plus Detection System (HRP) and the chromogen DAB (diaminobenzidine). Finally, cell nuclei were stained with hematoxylin.

### 2.10. Data Analysis

Data are expressed as the mean and standard deviation. For comparisons between groups, a two-tailed t-test was performed using Excel 2016, and a *p*-value < 0.05 was considered statistically significant. Image quantification was performed using Image J 1.52v software. For qualitative ELISA tests, the formula (MEAN + 3 × SD) for the negative controls was used to report positive tests [25].

## 3. Results

### 3.1. Purification of P1

**Figure 2:**
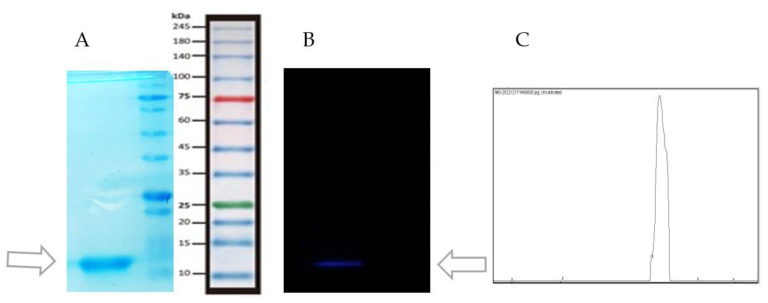
(**A**) A 12 kDa band was detected on SDS polyacrylamide 15% gel. (**B**) P1 protein with His-Tags interacts with anti-His monoclonal antibody in Western blot analysis. (**C**) Plot of the 12 kDa band in Western blot analysis, created with Image J (2.4% area).

The results showed that the P1 gene was overexpressed by the addition of 1 mM IPTG to BL21 culture medium, and was successfully purified and concentrated by the Nickle and 10 kDa Amicon columns. The protein was soluble in a 4 M urea solution at pH 7.3. In SDS-PAGE electrophoresis, a 12 kDa pure protein band was detected, which was proportional to the expected P1 molecular weight (Figure 2A). It also interacted with the anti-His monoclonal antibody, which is representative of the presence of His-Tags in the P1 protein (Figure 2B). His-Tags were added to the P1 protein when it was expressed in the vector PET-28-a.

### 3.2. Construction of pAK8-P1 Phagemid and Recombinant M13 Phage

**Figure 3:**
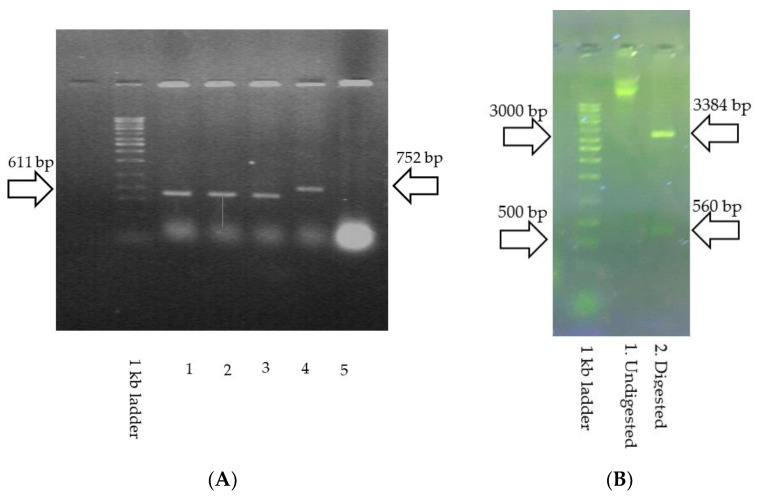
Construction of the pAK8-P1 phagemid. (**A**) Colony PCR with universal M13 primers: A 611 bp product is amplified in bacteria transformed with pAK8-P1 phagemid (lanes 1 to 3). Colony PCR on bacteria containing the pAK8 phagemid with unrelated inserts resulted in a 752 bp product (positive control for M13 phagemid, lane 4), and no amplification was observed in non-transformed bacteria (negative control, lane 5). (**B**) Restriction site mapping: Undigested phagemid was seen in lane 1. Digestion of pAK8 phagemid with EcoRI and HindIII resulted in 2560- and 3384-nucleotide DNA fragments, indicating the P1 gene’s insertion into the pAK8 phagemid, according to SnapGene analysis.

To confirm the insertion of the P1 gene into the pAK8 phagemid, different methods, including colony PCR, restriction enzyme mapping, and Sanger sequencing were performed. The 611 bp product of colony PCR with M13 universal primers on TG1-transformed cells was indicative of the insertion of the P1 gene into the pAK8 phagemid (Figure 3A). Digestion of the pAK8-P1 purified phagemid with the EcoRI and HindIII restriction enzymes yielded 2 DNA fragments, 560 and 3384 bp long, as predicted by the SnapGene software (Figure 3B). The Sanger sequencing result confirmed the presence of the gene in the correct orientation within the construct.

Infection of pAK8-P1-bearing TG1 bacteria with M13KO7 helper phages, followed by a PEG-NaCl precipitation step, resulted in the production of ~10^12^ pfu/mL of recombinant phage.

### 3.3. P1 Display on Recombinant M13 Phage

**Figure 4:**
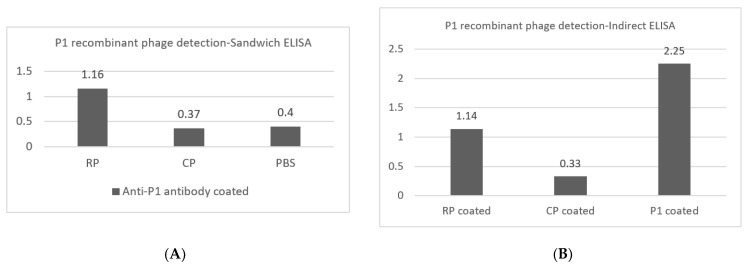
P1 display on recombinant M13 phage. (**A**) Sandwich ELISA: Coated anti-P1 antibodies interacted with P1 displayed on M13, and were then detected by monoclonal HRP-conjugated anti-M13 antibodies. CP and PBS were negative controls with optical densities below 0.4. (**B**) Indirect ELISA: Anti-P1 antibody at a dilution of 1:4000 reacted with P1 (positive control) and the recombinant phages (test), with the OD of the antibodies’ interactions with helper phages (negative control) being 0.33. *Y*-axis indicates optical density. Abbreviations: RP: P1 recombinant phage, CP: control phage, PBS: phosphate buffer saline.

Here, the presence of the P1 protein on the phage was checked by two qualitative ELISA methods. In the sandwich ELISA for the phage, recombinant phages displaying P1 reacted with purified anti-P1 antibodies and were visualized with anti-M13 HRP-conjugated antibodies at an optical density of 1.16. Control phages that did not display P1 had OD values of 0.37 (Figure 4A).

In indirect ELISA, anti-P1 antibodies detected P1 on recombinant phages and 1 µg purified P1 coated in the ELISA wells, and interacted with secondary anti-rabbit HRP conjugated antibodies (Figure 4B). The OD, which was detected in the wells coated with P1, was higher than in the wells with the recombinant phage. This could be due to the lower number of P1 proteins present on the M13 phage compared with 1 microgram of coated purified P1.

### 3.4. ACE2 Interaction with the P1-pVIII Fusion Protein

**Figure 5:**
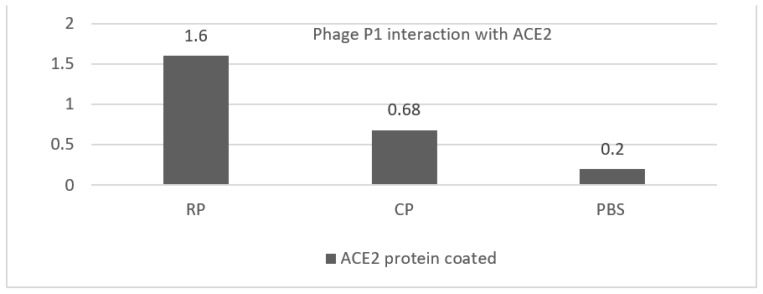
Interaction of P1 protein, displayed on bacteriophage pVIII, with ACE2 protein coated in ELISA wells. CP and PBS are negative control samples. The mean OD in the negative control wells was 0.44, with a standard deviation of ±0.27. An optical density >1.2 was considered positive (mean of negative control + 3*SD). *Y*-axis indicates the optical density. Abbreviations: RP: P1 recombinant phage, CP: control phage, PBS: phosphate buffer saline, ACE2: angiotensin-converting enzyme 2.

**Figure 6:**
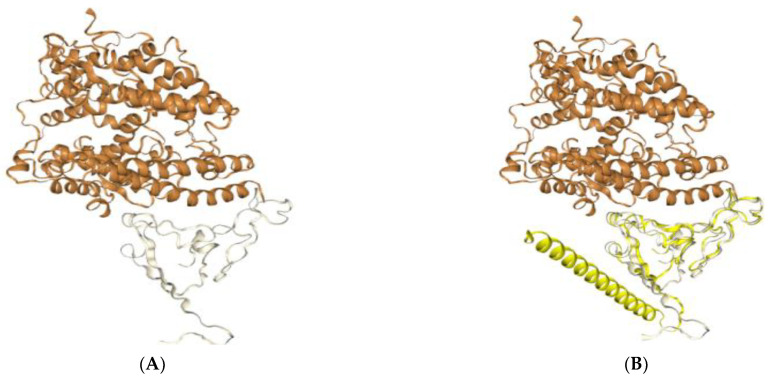
Docking result of the P1-pVIII fusion protein with the ACE2 receptor. The modeled structure is available on the PDB database (PDB ID: 6M0J). (**A**) The structure of the SARS-CoV-2 spike receptor-binding domain (shown in gray) bound to ACE2 with PDB ID 6M0J (brown color). (**B**) Docking result of the Hdock server indicating the possible interaction of the 157 amino acid P1-pVIII fusion protein (shown in yellow) with the ACE2 receptor (docking score −205.4, RMSD: 36.8).

Because the truncated P1 protein is RBD-based and contains the entire receptor-binding motif (RBM) of the spike protein, we hypothesized that there would be an interaction between ACE2 coated in ELISA wells and our recombinant P1 phage when P1 was displayed on the recombinant phages. The optical density of the wells where P1 recombinant phages were added was higher in comparison with the control phages (OD: 0.6) and PBS buffer (OD: 0.2), which may indicate an interaction between phage P1 and ACE2 (Figure 5). This confirmed the in silico docking data on the fusion of the P1-pVIII protein with ACE2, performed using the Hdock server (Figure 6).

### 3.5. Analysis of Animals Immunization against Phages

**Figure 7:**
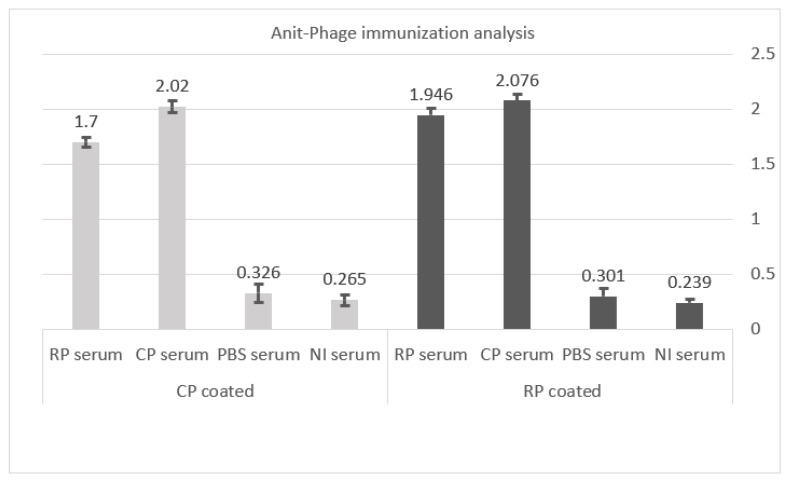
Immunization of mice against phage particles. Anti-phage antibodies were detected in both phage-inoculated groups of mice compared with the PBS and NI mice groups (*p*-value < 0.05). *Y*-axis indicates optical density. Abbreviations: RP: P1 recombinant phage, CP: control phage, PBS: phosphate buffer saline, NI: non-injected.

In this experiment, recombinant or helper phages were coated. Anti-phage IgG was detectable in the groups inoculated with both recombinant and helper phages, but the optical densities recorded from PBS-inoculated and non-injected mice serums (control negatives) were below 0.3. With a *p*-value < 0.05, there was a statistical difference between the phage-inoculated groups and the negative control groups (Figure 7).

**Figure 8:**
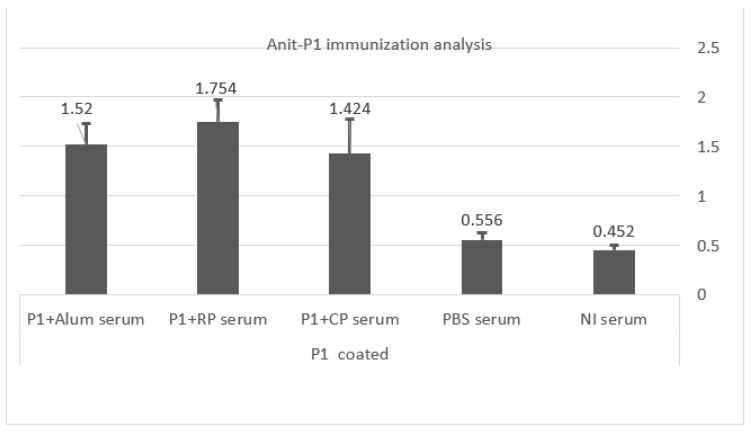
Immunization of mice against P1 protein: Anti-P1 antibodies increased in all mice immunized with P1+ recombinant/helper phage and in the group receiving P1 + Alum. There was a significant difference between the P1-receiving groups compared with the negative control groups (non-injected mice and PBS-inoculated group). *Y*-axis indicates optical density. Abbreviations: RP: P1 recombinant phage, CP: control phage, PBS: phosphate buffer saline, NI: non-injected.

Despite the display of P1 protein on recombinant M13, anti-P1 IgG did not increase to the detection level in the recombinant phage-inoculated group compared with the negative control groups (mice inoculated with control phage and PBS buffer and non-inoculated mice). However, mice receiving the P1 protein, along with the recombinant P1 phage or helper phage and alum adjuvant, all showed an increase in anti-P1 antibody titer compared to PBS-injected and non-injected mice, with the P1+ recombinant phage group showing the highest value (*p*-value < 0.05) (Figure 8). There was no significant difference between the P1-vaccinated groups. Inoculation of P1 without adjuvant was not performed in our study because we supposed that the administration of a purified protein in isolation would result in a weak or short-lasting immune response [26].

**Figure 9:**
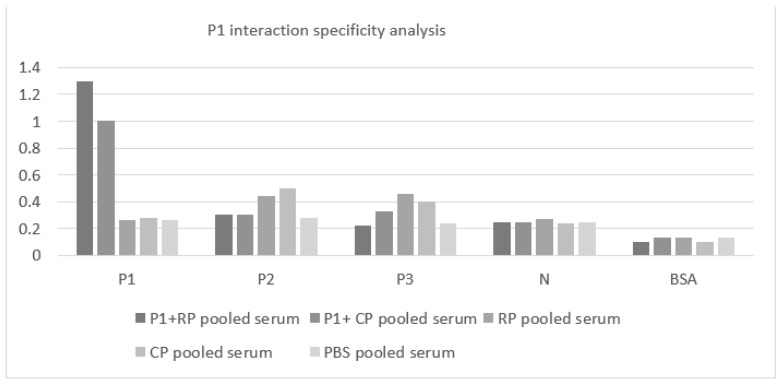
Analysis of the specificity of the P1 interaction: Groups of mice receiving P1+ recombinant/helper phages were immunized against the P1 protein and not against other unrelated proteins (P2, P3, N, and BSA). *Y*-axis indicates optical density. Abbreviations: RP: P1 recombinant phages, CP: control phage, PBS: phosphate buffer saline, NI: non-injected, BSA: bovine serum albumin.

Moreover, the pooled sera of P1+ recombinant/helper phages specifically interacted with P1 compared with other nonspecific proteins, i.e., P2, P3, N, and BSA (Figure 9).

### 3.6. Detection of Lymphocyte T CD4^+^ and CD8^+^ in Lung Tissues of Mice

**Figure 10:**
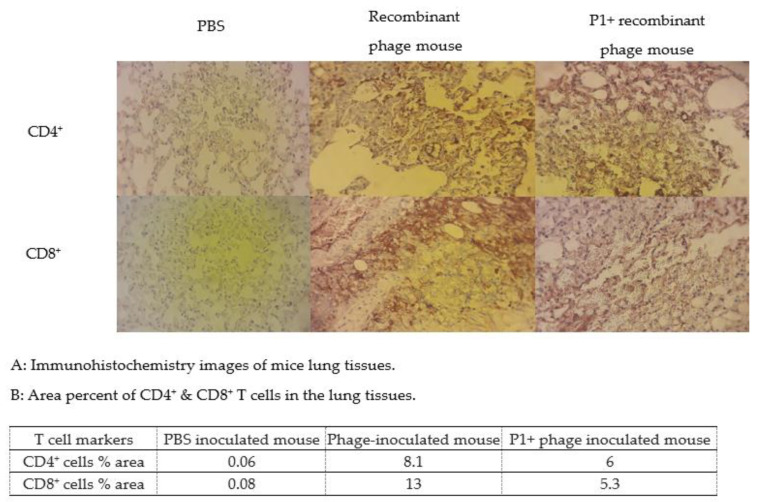
(**A**) Detection of CD4^+^ and CD8^+^ T cells in mouse lungs by immunohistochemistry analysis: The brown color in the images of mice that received recombinant phages and mice that received P1+ recombinant phages is indicative of the presence of CD4^+^ and CD8^+^ T cells in the lungs (5 to 13 percent area), whereas almost no color change was seen in the mouse injected with PBS (% area < 0.08). (**B**) Quantitative analysis of the area with T CD4^+^ and T CD8^+^ cells was performed using Image J software and reported as percent area for each image in the table.

The lung is the most affected organ in COVID-19 [27], and the presence of T cells at this site as representatives of cell-mediated immunity may lead to control of the viral infection through lymphocyte B activation, inflammatory cytokine production, and direct virus elimination [28]. Brown staining of the lung tissue from mice inoculated with P1 + recombinant P1 phage and recombinant P1 phage alone indicated the presence of lymphocyte T CD4^+^/CD8^+^. Almost no color change was seen in the lung tissue of the mouse that was not injected (Figure 10A). According to Image J analysis, approximately 5 to 13 percent of CD4^+^ and CD8^+^ T cells were present in the lung tissues of the mice with P1 phage and P1 + P1 phage inoculation (Figure 10B).

## 4. Discussion

The ease of manipulation of the phage genome, its inexpensive production, its inherent ability to stimulate the immune system, and its flexibility in modes of administration, combined with its stability at ambient temperatures and over a wide pH range, make the phage vaccine an ideal platform for vaccine development, as large-scale production of the vaccine, route of administration, transportation, and storage are all essential issues with which scientists are concerned.

Administration of the truncated S P1 with Freund’s adjuvant was effective in activation of the cellular and humoral immune responses, resulting in neutralizing antibodies against SARS-CoV-2 virus without histological side effects [21]. In this study, we displayed this 101-amino acid protein on the M13 bacteriophage to determine whether it would be able to stimulate the immune system of mice and neutralize the virus. Our results showed that, regardless of the display of P1 on the recombinant phage protein VIII, its interaction with the recombinant truncated ACE2 protein expressed in HEK293 (both in silico and in vitro by ELISA experiment) and the activation of CD4^+^ and CD8^+^ T cells, the humoral immune response was mainly directed against the phage nanoparticle itself and not against the displayed antigen P1. According to the literature, phage pVIII allows for the display of a larger number of peptides per phage particle, although the peptide size is limited to 6–20 amino acids. Insertion of a larger peptide/protein DNA sequence into a phagemid results in inefficient phage assembly and lower peptide/protein display on the pVIII. It is said that when using a phagemid system, a 23 kDa protein is displayed on pVIII on average less than a single copy per phage particle [29]. Given this size limitation, several research teams have developed recombinant phage vaccines containing short peptides on pVIII instead of larger protein units. Here are some examples of these effective vaccines: 1. Sartorius et al. engineered fd filamentous phages to display 8 and 9 amino acid peptides for stimulation of the antitumor CTL (cytotoxic T lymphocyte) response [30]; 2. another group presented a 15-mer glycoprotein G epitope on the fd protein VIII to produce an antiviral vaccine [31]; 3. in another study, a CTL response was induced by a recombinant M13 vaccine with 12-mer hepatitis B virus peptides on its major protein [32]; and 4. fd phages expressing tumor-specific epitopes of melanoma antigen A1 (161–169) on pVIII were another potential anti-cancer phage vaccine developed by Fang J. et al. [33].

On closer inspection, phage vaccines have not always favored researchers. In a study attempting to search for a potential vaccine against COVID-19, 6 epitopes of the S protein, ranging in size from 9 to 26 amino acids, were displayed on the main phage protein (~300 copies), and animals were inoculated with 10^9^ particles of these phages at two intervals. Although the animals in all 6 groups were immunized against the bacteriophages, anti-S IgG was detected only in the group receiving phages with epitope 4 (10 amino acids). They suggested that the conserved epitope 4 might elicit a strong and specific humoral anti-S immune response because it recapitulates the near-native structure when expressed on the protein VIII based on the analysis of molecular dynamics [9].

In 2015, Asadi M et al. displayed a 15-amino acid EGFR (epidermal growth factor receptor) mimotope on the M13 major protein using the pAK8 phagemid, and observed a small (only 15% difference in OD) anti-EGFR mimotope antibody in immunized mice compared with the controls. There was no protective and therapeutic antitumor activity in vivo, likely due to the low anti-mimotope antibody titer. However, the phage particles themselves were able to reduce the tumor growth of a Lewis lung carcinoma compared with the negative control receiving PBS [34]. This anti-tumor properties of bacteriophages have been proposed previously by other research groups [35]. It has been hypothesized that the expression of tandem repeats of peptides on the phage surface might be the key to sufficient and effective stimulation of the immune system [34]. Following this assumption, Javanmardi M. et al. constructed two recombinant phage particles with the same pAK8 phagemid, one displaying a twelve-amino acid mimotope (termed 1M) and the other expressing three repeats of 1M (thirty-six amino acids, termed 3M). While no significant difference was found between the antitumor effects of the recombinant 1M and 3M phages in the preventive or therapeutic groups, the mice immunized with the 3M phage showed prophylactic and therapeutic effects, as well as prolonged survival compared with the PBS group [36]. These results may highlight the importance of the ratio of peptide/protein display per bacteriophage in the stimulation of the immune system. In an attempt to increase the exposure and accessibility of the EGFR mimotope to the immune system, Asadi M. and colleagues undertook research in which they modified the M13 phage genome to display a 148-amino acid protein called EM-L2 (the sequence of the EGFR mimotope containing the L2 extracellular domain of EGFR) on pVIII. These phages were successful in immunizing animals and treating tumors by activating both arms of the cellular immune system, but they were inefficient in preventing tumor formation [37]. Surprisingly, in 1997, Bastien N. et al. constructed recombinant fd phages that have disease-specific protective epitopes (15 amino acids) of glycoprotein G of human RSV (respiratory syncytial virus) on their protein III [38] (only five copies are present on the fd bacteriophage [39]). A high level of RSV-specific antibodies was detected in the mouse serum immunized with these phages, indicating that the mice were protected from RSV infection [38]. All in all, it can be concluded that the nature of the antigen, its length, and the number of expressions on the bacteriophage are crucial factors for the efficacy of a vaccine.

In our second strategy, we injected 50 µg of purified P1 with recombinant or helper phages in 2 different groups of animals at 2 intervals. We observed that the anti-P1 antibody titer increased after the second administration dose, whereas the first one was higher. In addition, the results of the IHC assay showed that both CD4^+^ and CD8^+^ T lymphocytes were present in the lung tissues of the animals. These results suggest the importance of protein concentration in determining the extent of immune system stimulation. With this in mind, researchers have previously used T4 SOC and HOC proteins to display multiple copies of longer proteins, of which there are 810 and 155 copies per T4 capsid, respectively. In one study, scientists fused the mVEGFR (vascular endothelial growth factor receptor) gene to the C-terminus of the T4-Soc gene, resulting in a recombinant phage that inhibited tumor angiogenesis in a mouse lung cancer model [40]. Wu J. and colleagues engineered the T4 phage to simultaneously display the CSFV (classical swine fever virus) 123 amino acid mE2 antigen of CSFV (classical swine fever virus) at SOC and the 371 amino acid full-length CSFV E2 antigen at HOC. This dual-site display phage successfully enhanced the immune responses of the mice to the CSFV antigens [41]. In another study on the recombinant bacteriophage Lambda, antitumor immunity in hepatocellular carcinoma was induced by Lambda phages expressing human ASPH (aspartate β-hydroxylase)-derived proteins on their GpD proteins [42] (approximately 405 to 420 copies are present per phage particle [43]).

In addition, the researchers used other techniques to increase the number of proteins displayed on the phages, rather than just altering the phage genomes to display the desired antigen in vivo. In our case, for the COVID-19 disease, a T4-CoV-2 nanovaccine was produced by conjugating purified spike trimer protein to T4 phage (~100 trimers per T4 phage). On days 0 and 21, animals were administered different doses of the S-trimer antigen: 0.8, 4.8, and 20 µg, corresponding to 10^10^, 6 × 10^10^, and 2.5 × 10^11^ phage particles, respectively. This nanovaccine was effective in activating cellular and humoral immunity and producing virus-neutralizing antibodies. All immunized mice challenged with SARS-CoV-2 survived for 21 days after the challenge [17]. The other group engineered the coat protein of bacteriophage MS2 to display multiple copies of the S protein using the streptavidin–biotin conjugation method. Golden Syrian hamsters received MS2-SA virus-like particles (VLPs) containing 60 µg of the S protein on a one-time basis. High levels of neutralizing antibodies were detected in the sera of the animals, protecting them from the COVID-19 virus and preventing the appearance of the infectious virus in the lungs [14]. These results were similar to the research findings of Guo Y. et al. who created a chimeric VLP of phage Qbeta (Qβ) that had universal epitopes of SARS-CoV-2 and generated specific antibodies with virus-neutralizing activities [15].

Researchers have advanced a step further and used a phage-based DNA vaccine as a gene carrier so that the task of protein expression and folding was assigned to the host cells [44]. Clark J.R. et al. applied a lambda phage DNA vaccine expressing hepatitis B HBsAg and demonstrated its disease-protective potential compared with the commercially available recombinant protein vaccine, Engerix B [45]. Another group used AAVP (adeno-associated virus phage) technology to deliver the full SARS-CoV-2 spike protein gene for expression in host cells. They detected a specific humoral anti-S and phage response in the immunized mice [9].

Some studies have suggested that intranasal administration of the phage vaccine against COVID-19, together with the targeted application of phages to lung epithelial cells, is locally effective in enhancing the immune response against SARS-CoV-2 [9,17]. These factors should not be ignored when evaluating the efficacy of phage vaccines.

Altogether, the preparation and precipitation of the recombinant P1 phage were simple, rapid, and unproblematic compared with the preparation and purification of the P1 protein vaccine. The P1 protein produced neutralizing antibodies against the virus and interacted with antibodies in the serum of COVID-19 patients, but the production process was more tedious and required special attention to temperature and pH [21]. Because the recombinant phage was unable to elicit a virus-neutralizing anti-P1 immune response in the mice, it failed to qualify as a good vaccine against COVID-19, and should be redeveloped and improved.

## 5. Conclusions

Considering the potential of phage vaccines, it can be emphasized that the copy numbers of peptides/proteins displayed on the phages represent a key factor for the success of the phage vaccine in preventing or treating diseases. We hypothesize that the development of an ideal phage-based vaccine will depend on tactful answers to the following questions: Which antigen on which type of phage and expression system should be produced, and how would it be transferred into the host body?

In addition, phages can be used alone or conjugated with antigens to activate the immune system and act as adjuvants. They can be injected together with antigens, whether or not they are conjugated to the desired antigen. Thus, phage technology has potential in vaccine development, but requires further investigation in terms of the safety, size, quantity, and weight of antigen expression on the phage surface, etc.

## Data Availability

Not applicable.
